# Demethyleneberberine Alleviates Pulmonary Fibrosis through Disruption of USP11 Deubiquitinating GREM1

**DOI:** 10.3390/ph17030279

**Published:** 2024-02-22

**Authors:** Chuang Ge, Mengsheng Huang, Yanhong Han, Chang Shou, Dongyin Li, Yubin Zhang

**Affiliations:** State Key Laboratory of Natural Medicines, Department of Biochemistry, China Pharmaceutical University, #639 Longmian Avenue, Jiangning District, Nanjing 211198, China; 3120030155@stu.cpu.edu.cn (C.G.); huangmengsheng@stu.cpu.edu.cn (M.H.); 3220030533@stu.cpu.edu.cn (Y.H.); 3221031051@stu.cpu.edu.cn (C.S.); 3222031004@stu.cpu.edu.cn (D.L.)

**Keywords:** DMB, GREM1, USP11, pulmonary fibrosis, ubiquitination

## Abstract

Background: Idiopathic pulmonary fibrosis (IPF) is a fatal and chronic interstitial lung disease. Intricate pathogenesis of pulmonary fibrosis and only two approved medications with side effects and high cost bring us the challenge of fully understanding this lethal disease and urgency to find more safe and low-cost therapeutic alternatives. Purpose: Demethyleneberberine (DMB) has been demonstrated to have various anti-inflammatory, antioxidant, antifibrosis and anti-cancer bioactivities. The objective of this study was to evaluate the effect of DMB on pulmonary fibrosis and investigate the mechanism. Methods: Bleomycin (BLM)-induced pulmonary fibrosis was established in mice to evaluate the antifibrotic effect of DMB in vivo. A549 and MRC5 cells were used to evaluate the effect of DMB on epithelial–mesenchymal transition (EMT) and fibroblast–myofibroblast transition (FMT) in vitro. High throughput sequencing, biotin–avidin system and site-directed mutagenesis were applied to explore the mechanism of DMB in alleviating pulmonary fibrosis. Results: DMB alleviated BLM-induced pulmonary fibrosis in vivo by improving the survival state of mice, significantly reducing pulmonary collagen deposition and oxidative stress and improving lung tissue morphology. Meanwhile, DMB was demonstrated to inhibit epithelial–mesenchymal transition (EMT) and fibroblast–myofibroblast transition (FMT) in vitro. High throughput sequencing analysis indicated that GREM1, a highly upregulated profibrotic mediator in IPF and BLM-induced pulmonary fibrosis, was significantly downregulated by DMB. Furthermore, USP11 was revealed to be involved in the deubiquitination of GREM1 in this study and DMB promoted the ubiquitination and degradation of GREM1 by inhibiting USP11. Remarkably, DMB was demonstrated to selectively bind to the Met776 residue of USP11, leading to disruption of USP11 deubiquitinating GREM1. In addition, DMB presented an equivalent antifibrotic effect at a lower dose compared with pirfenidone and showed no obvious toxicity or side effects. Conclusions: This study revealed that USP11/GREM1 could be a potential target for IPF management and identified that DMB could promote GREM1 degradation by inhibiting USP11, thereby alleviating pulmonary fibrosis.

## 1. Introduction

Idiopathic pulmonary fibrosis (IPF) is a fatal and chronic interstitial lung disease in which myofibroblast foci formation and massive amount of extracellular matrix (ECM) components’ deposition eventually leads to the destruction of lung function [1,2]. The pathogenesis of IPF remains elusive, involving repeated injury and abnormal repair of alveolar epithelium, abundant secretion of various cytokines, epithelial–mesenchymal transition (EMT), activation and differentiation of fibroblasts, as well as crosstalk between fibroblasts and epithelial cells [3,4,5,6,7]. Despite ongoing efforts in the diagnosis and treatment of IPF [8], there remains a significant lack of effective drugs. Only pirfenidone and nintedanib are approved medications for IPF therapy in clinic and both of them have been shown to delay IPF progression, but gastrointestinal side effects and transaminitis are reported in both treatments, and dermatological side effects exist in pirfenidone treatment [9,10]. Therefore, it is urgent to develop other therapeutic alternatives.

Bone morphogenetic proteins (BMPs) belong to the TGFβ superfamily and play a role in countering TGFβ signaling in many physiological processes [11]. BMP signaling contributes to antifibrotic effects against TGFβ1 signaling but is usually repressed by BMP antagonists in pulmonary fibrosis [12,13]. Gremlin-1 (GREM1) belongs to the family of BMP antagonists and is upregulated in mice and clinical patients with pulmonary fibrosis [14,15,16]. An increasing number of studies have demonstrated that GREM1 has a profibrotic effect and contributes to pulmonary fibrosis [15,16,17,18], renal fibrosis [19] and colonic fibrosis [20].

Protein degradation in eukaryotes is largely mediated by the ubiquitin proteasome system (UPS). Ubiquitination is a dynamic process in which deubiquitinating enzymes (DUBs) can remove ubiquitin chains from polyubiquitinated substrates [21,22]. USP11 is a part of the most common family of DUBs, the ubiquitin-specific protease (USP) family. USP11 participates in the deubiquitination and stabilization of multiple proteins, including the type I TGFβ receptor (ALK5) [23], cellular inhibitor of apoptosis protein-2 (cIAP2) [24] and IκBα [25]. Some investigations have suggested that USP11 is implicated not only in tumorigenesis [26,27] but also in the progression of pulmonary fibrosis in recent studies [28,29]. However, no research has reported the mechanism underlying GREM1 ubiquitination and the interaction between USP11 and GREM1 so far.

Demethyleneberberine (DMB), a natural product, is a component from the rutaceous plant *Cortex Phellodendri Chinensis* (CPC) with anti-inflammatory, neuroprotective, antioxidant and antimicrobial properties [30,31,32,33]. DMB can be synthesized by thedemethylation of berberine in our laboratory and previous studies have reported that DMB could alleviate alcoholic or non-alcoholic fatty liver disease, hepatic fibrosis, inflammatory bowel disease and Pseudomonas aeruginosa-induced acute pneumonia [34,35,36,37,38,39]. The intervention of DMB on pulmonary fibrosis has not been investigated.

Here, our study aimed to investigate the therapeutic potential of DMB in alleviating pulmonary fibrosis, as well as to explore the underlying mechanisms. Our work illustrated that DMB alleviated BLM-induced pulmonary fibrosis and inhibited EMT and FMT. High throughput sequencing illustrated that DMB significantly decreased GREM1 in the lungs of mice. Further studies revealed that DMB promoted GREM1 degradation via Ub/proteasome pathway through disruption of USP11 deubiquitinating GREM1.

## 2. Results

### 2.1. DMB Alleviates BLM-Induced Pulmonary Fibrosis in Mice

**Figure 1:**
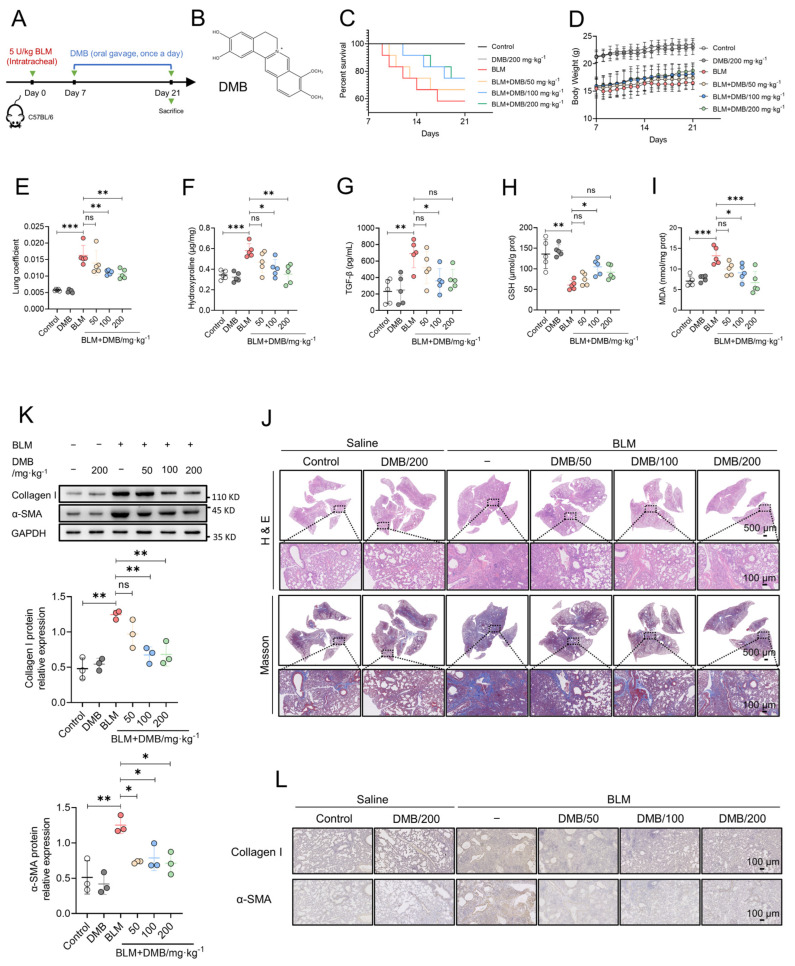
DMB alleviates BLM-induced pulmonary fibrosis in mice. (**A**) The diagram of animal model construction and treatment regimen with DMB. (**B**) The chemical structure of DMB. (**C**) Survival percentage of mice. (**D**) Body weight. (**E**–**I**) Lung coefficient (**E**), the level of hydroxyproline (**F**), TGF-β1 (**G**), GSH (**H**), MDA (**I**) in the lung tissues of mice (n = 5). (**J**) Representative images of HE and Masson staining of the whole lungs in different groups. Scale bar, 500/100 μm. (**K**) α-SMA and collagen I expression in the lung sections of mice in the indicated groups were analyzed by Western blot. (**L**) Representative images of IHC staining (α-SMA and collagen I) of the lungs 21 days after BLM challenge. Scale bar, 100 μm. Data are presented as the mean ± SD. * *p* < 0.05, ** *p* < 0.01 and *** *p* < 0.001, ns, not significant.

The treatment efficacy of DMB on pulmonary fibrosis was investigated by carrying out BLM-induced pulmonary fibrosis in mice, which were administered DMB (50, 100, 200 mg/kg) for 14 days (Figure 1A,B). DMB improved the survival state of mice (Figure 1C,D) and decreased the lung coefficient (Figure 1E). The content of hydroxyproline and TGF-β1 in the lung tissues of mice were significantly reduced after treatment with DMB (Figure 1F,G). When pulmonary fibrosis occurs, excessive reactive oxygen species (ROS) cause damage to alveolar epithelial cells [40]. Treatment with DMB increased the glutathione (GSH) level and reduced the malonaldehyde (MDA) level in the lung tissues (Figure 1H,I), which suggested that the oxidative stress was relieved in the lungs of mice treated with BLM. Histopathological examination revealed that DMB treatment reduced collagen deposition and improved lung tissue morphology compared with no treatment (Figure 1J). A consistent result also appeared in Western blot and immunohistochemistry analyses, which indicated that collagen I and α-SMA were reduced by DMB (Figure 1K,L). Overall, our study demonstrated that DMB significantly reduced pulmonary collagen deposition, relieved oxidative stress and improved lung tissue morphology, effectively alleviating pulmonary fibrosis in mice.

### 2.2. DMB Inhibits Epithelial–Mesenchymal Transition and Fibroblast–Myofibroblast Transition

**Figure 2:**
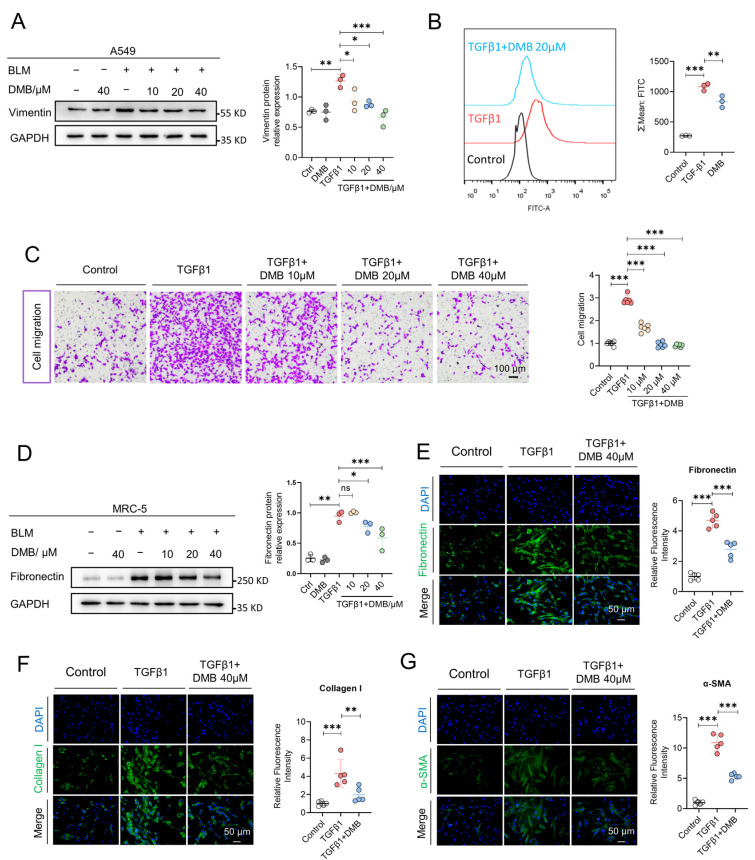
DMB inhibits epithelial-mesenchymal transition and fibroblast-myofibroblast transition in vitro. (**A**) Vimentin expression in A549 cells treated with TGF-β1 (5 ng/mL) and DMB (10, 20 40 μM) was analyzed by Western blot. (**B**) Flow cytometry was applied to analyze oxidation resistance of DMB with DCFH-DA (n = 3). Scale bar, 50 μm. (**C**) Cell migration was detected by transwell assay in the indicated groups (n = 6). Scale bar, 100 μm. (**D**) Fibronectin expression in MRC-5 cells treated with TGF-β1 (5 ng/mL) and DMB (10, 20 40 μM) was analyzed by W estern blot. (**E**–**G**) Representative images of immunofluorescence staining of fibronectin (**E**), collagen I (**F**) and α-SMA (**G**) in MRC-5 cells treated with TGF-β1 (5 ng/mL) and 40 μM DMB (n = 5). Blue, DAPI; green, collagen I, fibronectin, α-SMA. Scale bar, 50 μm. Data are presented as the mean ± SD. * *p* < 0.05, ** *p* < 0.01 and *** *p* < 0.001, ns, not significant.

Activated myofibroblasts deposit excessive amounts of extracellular matrix (ECM) including fibronectin and collagen I contributing to fibrosis progression. These myofibroblasts are mainly derived from fibrocytes, resident fibroblasts and the epithelial–mesenchymal transition [41,42]. We assessed the effect of DMB on inhibiting the epithelial–mesenchymal transition and fibroblasts’ transition into myofibroblasts in vitro. Vimentin, one of the key biomarkers of EMT and usually expressed in mesenchymal cells, was upregulated when treated with TGF-β1 and markedly reduced after DMB treatment (Figure 2A). In the lung tissues of mice treated with BLM, DMB relieved oxidative stress as shown in in vivo study. Consistent results were obtained in flow cytometry in vitro, which indicated that DMB reduced the ROS level in cells (Figure 2B). Moreover, as shown in a transwell migration assay (Figure 2C), DMB inhibited the migration of epithelial cells. We next investigated whether DMB could inhibit fibroblast–myofibroblast transition and reduce ECM production. Collagen I and fibronectin levels were reduced in MRC-5 cells following treatment with DMB, while α-SMA was also downregulated (Figure 2D–G). These results suggested that DMB significantly inhibited EMT and FMT, reduced ECM production and exhibited oxidation resistance in vitro, consistent with the findings observed in vivo.

### 2.3. DMB Promotes the Degradation of GREM1 byt Ub/Proteasome Pathway

**Figure 3:**
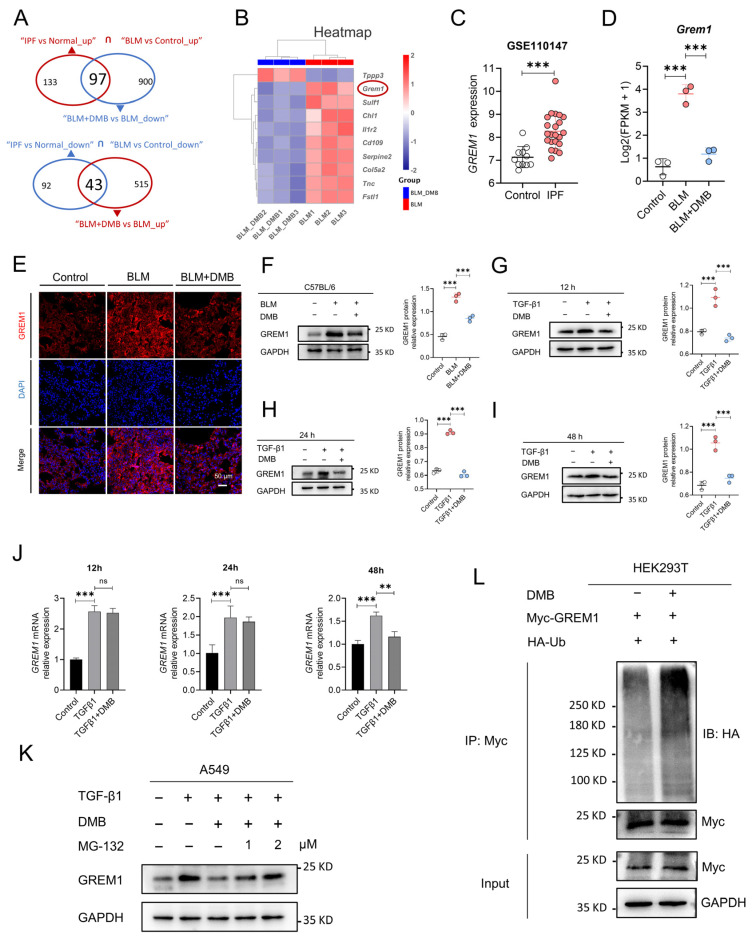
DMB promotes GREM1 degradation through Ub/ proteasome pathway. (**A**) RNA-seq analysis of control, BLM and BLM + DMB groups. Differential expression genes were analyzed between people without IPF and IPF patients from “GSE110147”. Common differential expression genes were obtained between homo sapiens and mice and 140 (97 + 43) genes were significantly regulated by DMB. (**B**) Heatmap of 140 (97 + 43) genes. (**C**) GREM1 expression between IPF and control samples. (**D**) GREM1 expression between control, BLM and BLM + DMB samples (n = 3). (**E**) Immunofluorescence staining of GREM1 in the lung tissue sections of mice. Scale bar, 50 μm. Blue, DAPI; red, GREM1. (**F**–**I**) GREM1 protein expression in the lung tissues of BLM-treated mice and TGF-β1 (5 ng/mL)-treated A549 cells with DMB treatment by Western blot. (**J**) GREM1 mRNA expression in A549 cells (n = 3). (**K**) GREM1 protein expression in A549 cells treated with TGF-β1 (5 ng/mL) and 40 μM DMB with or without MG-132 by Western blot. (**L**) 293T cells were transfected with Myc-GREM1 and HA-Ub with or without DMB. Anti-myc antibody was used to immunoprecipitate Myc-GREM1. Western blot was applied to analyze the precipitates. Data are presented as the mean ± SD. ** *p* < 0.01 and *** *p* < 0.001, ns, not significant.

RNA sequencing was performed to explore gene expression profiling to explore the mechanism by which DMB alleviated pulmonary fibrosis. In the lung tissues of the BLM group and the control group, 2070 genes were elevated and 1834 genes were downregulated. A total of 558 and 997 genes, respectively, were upregulated and downregulated between the BLM + DMB and BLM groups (Figure S1A–C). Meanwhile, the differentially expressed genes were analyzed between IPF patients and normal controls from Gene Expression Omnibus (GEO) database. A total of 2328 genes were upregulated and 1392 genes were downregulated between IPF patients and normal controls (Figure S1D,E). Furthermore, common gene expression patterns between BLM-treated mice and IPF patients illustrated 230 genes’ upregulation and 135 genes’ downregulation. However, the expression level of 97 genes from 230 genes and 43 genes from 135 genes were reversed by DMB (Figure 3A). Top 10 genes of above 140 genes (97 + 43) were shown in the heatmap (Figure 3B). GREM1, a profibrotic mediator, was upregulated in BLM-treated mice and IPF patients (Figure 3C,D) and DMB downregulated GREM1 protein expression level (Figure 3E,F). The same results were shown in the TGFβ1-induced A549 cells (Figure 3G–I). However, GREM1 mRNA expression level was only downregulated at 48 h after DMB treatment without change at 12 h and 24 h (Figure 3J), suggesting that DMB may also reduce GREM1 protein expression through other pathways. We focused on ubiquitin–proteasome system (UPS), the main pathway for protein degradation in eukaryotes [21,22]. The effect of DMB promoting GREM1 degradation was reversed by coincubation with the proteasome inhibitor MG-132 (Figure 3K). Furthermore, HA-Ub and Myc-GREM1 were transfected into 293T cells to detect the ubiquitination of GREM1 and DMB-accelerated GREM1 ubiquitination (Figure 3L). These results suggested that DMB significantly downregulated GREM1 expression, promoting GREM1 ubiquitination and degradation by the Ub/proteasome pathway.

### 2.4. USP11 Stabilizes GREM1 by Deubiquitination

**Figure 4:**
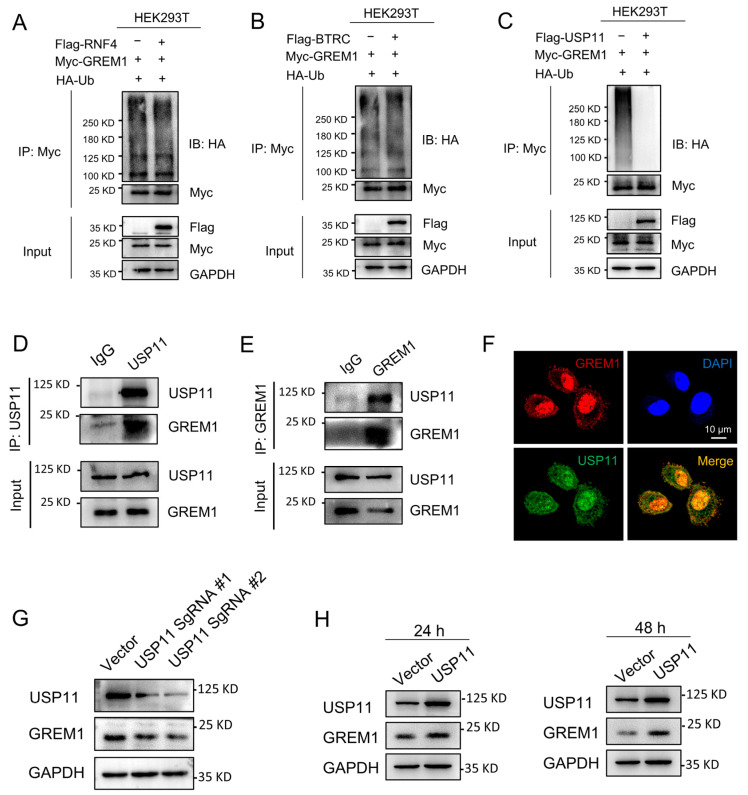
USP11 stabilizes GREM1 by deubiquitination. (**A**–**C**) 293T cells were transfected with Myc-GREM1, Flag-USP11, Flag-RNF4, Flag-BTRC and HA-Ub. Anti-myc antibody was used to immunoprecipitate Myc-GREM1. Western blot was applied to analyze the precipitates with anti-HA antibody. (**D**,**E**) Co-IP was applied to demonstrate the interaction of USP11 and GREM1 with anti-USP11 (**D**) or anti-GREM1 antibody (**E**). (**F**) Visualization of the localization of USP11 and GREM1 by immunofluorescence. Blue, DAPI; red, GREM1; green, USP11. Scale bar, 10 μm. (**G**) USP11 CRISPR/Cas9 constructs were transfected into A549 cells. Total protein from cell lysates was analyzed by Western blot. (**H**) Flag-USP11 constructs were transfected into A549 cells. Total protein from cell lysates was analyzed by Western blot.

Database UbiNET 2.0 recorded two E3 ubiquitin ligases, RNF4 and BTRC, that modified DAND5 (GREM3), another one of the DAN family. Additionally, the research reported that USP11 can deubiquitinate hybrid SUMO-ubiquitin chains to counteract RNF4 [43]. Therefore, we investigated whether RNF4, BTRC and USP11 were involved in the regulation of GREM1 ubiquitination. Results showed that USP11 overexpression reduced the ubiquitination of GREM1 and that TNF4 and BTRC overexpression had no effect on the ubiquitination of GREM1 (Figure 4A–C). USP11 is a part of the ubiquitin-specific processing protease (USP) family and is involved in the deubiquitination of type I TGFβ receptor (ALK5) to augment TGFβ signaling [23]. Recent studies have suggested that USP11 is involved in IPF progression [28,29]. Furthermore, the interaction of USP11 and GREM1 was investigated by co-IP assay, which was performed in the A549 cells. We found that GREM1 was detected by Western blot in the immunoprecipitates after USP11 was immunoprecipitated (Figure 4D). Consistently, USP11 was detected in the immunoprecipitates after GREM1 was immunoprecipitated (Figure 4E). In addition, immunofluorescent staining indicated colocalization of USP11 and GREM1 (Figure 4F). USP11 Knockdown contributed to a decrease in GREM1 expression level (Figure 4G), and USP11 overexpression resulted in an increase in GREM1 expression level (Figure 4H). Collectively, these findings demonstrated that USP11 stabilized GREM1 by deubiquitination.

### 2.5. DMB Directly Binds to USP11 and Inhibits Its Deubiquitination on GREM1

**Figure 5:**
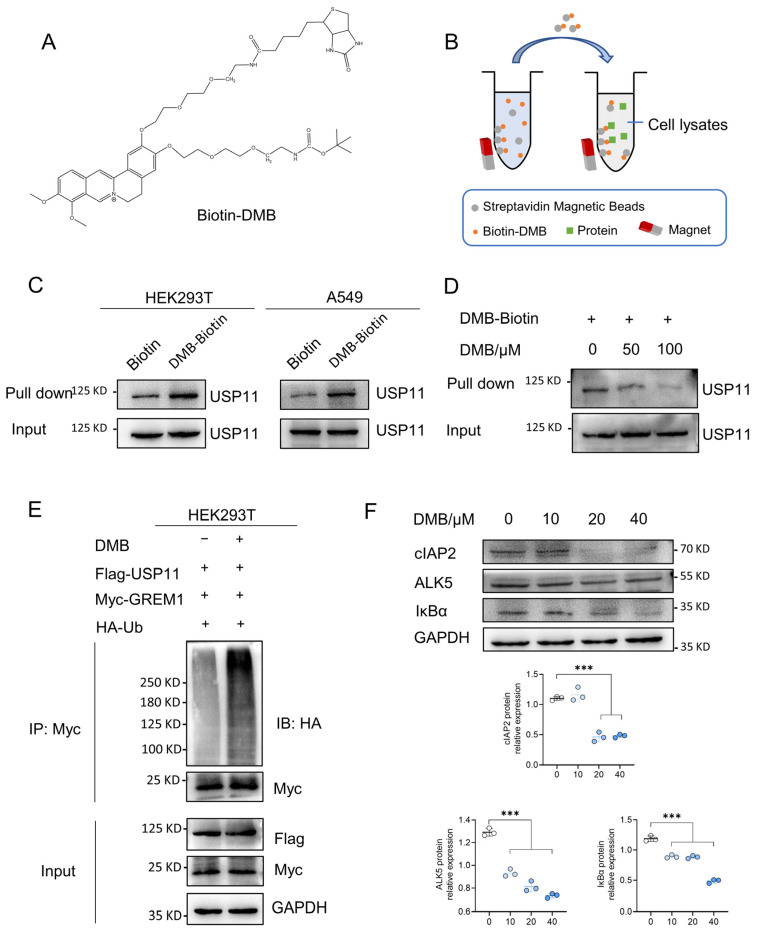
DMB binds to USP11 and inhibits its deubiquitination. (**A**) Structure of Biotin-DMB. (**B**) Diagram of Biotin-DMB binding to targeted protein. (**C**) Streptavidin magnetic beads coupled with Biotin-DMB were incubated with A549 and 293T cell lysates. Western blot was applied to analyze the precipitates with anti-USP11 antibody. (**D**) Unlabeled DMB competitively bound to USP11 with Biotin-DMB. (**E**) 293T cells were transfected with Myc-GREM1, Flag-USP11 and HA-Ub with or without DMB. Anti-myc antibody was used to immunoprecipitate Myc-GREM1. Western blot was applied to analyze the precipitates with anti-HA antibody. (**F**) DMB negatively regulates other substrates of USP11. Data are presented as the mean ± SD. *** *p* < 0.001.

The above studies revealed that DMB promoted the degradation of GREM1 by increasing its ubiquitination and USP11 could stabilize GREM1 by deubiquitination. We thus hypothesized that DMB facilitated the degradation of GREM1 by the disruption of USP11 deubiquitinating GREM1. It is necessary to reveal the direct binding of DMB to USP11 firstly. The biotin–avidin system was a preferred choice to detect DMB directly binding to USP11. We synthesized biotin-conjugated DMB (Biotin-DMB) (Figure 5A and Figure S2) and incubated Biotin-DMB with streptavidin magnetic beads. Subsequently, target proteins binding to DMB were pulled down by incubating streptavidin magnetic beads coupled with Biotin-DMB with 293T and A549 cell lysates (Figure 5B). Western blot was used to analyze the precipitates with an anti-USP11 antibody, which indicated that USP11 was detected in the precipitates (Figure 5C). Additionally, a competition binding assay revealed that unlabeled DMB decreased the binding of Biotin-DMB to USP11 (Figure 5D). These findings revealed that DMB directly bound to USP11. Furthermore, 293T cells were transfected with Flag-USP11, Myc-GREM1 and HA-Ub with or without DMB. DMB increased GREM1 ubiquitination, suggesting that DMB could inhibit the function of USP11 and promote GREM1 degradation (Figure 5E). To confirm this conclusion, we explored whether DMB promoted the degradation of some other proteins which were stabilized by USP11 and found that DMB also decreased the expression level of cIAP2, ALK5 and IκBα (Figure 5F). Taken together, DMB directly bound to USP11 and accelerated GREM1 degradation through the disruption of USP11 deubiquitinating GREM1.

### 2.6. Met776 of USP11 Is Critical for DMB Binding to USP11

**Figure 6:**
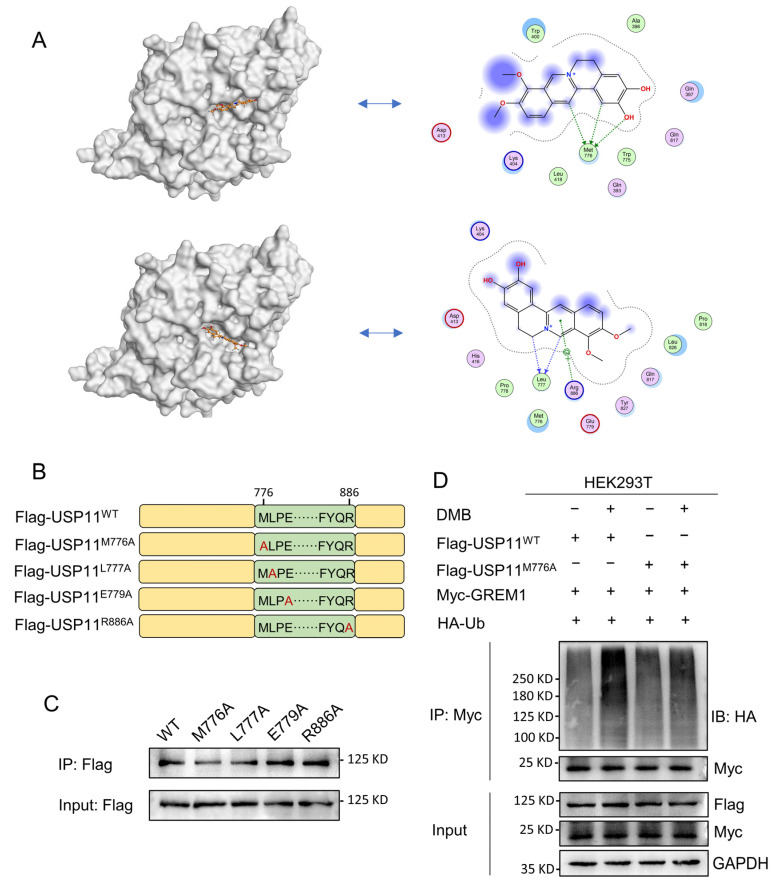
Met776 of USP11 is critical for DMB binding to USP11. (**A**) DMB could bind to catalytic domain of USP11 by molecular docking. (**B**) Schematic diagram of site-directed mutagenesis at Met776, Leu777, Glu779 and Arg886 residue with alanine (**A**). (**C**) 293T cells were transfected with wild-type Flag-USP11 and indicated mutant Flag-USP11 plasmid constructs. Streptavidin magnetic beads coupled with Biotin-DMB were incubated with 293T cell lysates. Western blot was applied to analyze the precipitates with anti-Flag antibody. (**D**) 293T cells were transfected with Myc-GREM1, HA-Ub, Flag-USP11^WT^, Flag-USP11^M776A^ with or without DMB. Anti-myc antibody was used to immunoprecipitate Myc-GREM1. Western blot was applied to analyze the precipitates with anti-HA antibody.

USP11 is a multiple domain protein consisting of DUSP, UBL and catalytic domain (Figure S3). We performed a molecular docking of DMB with the catalytic domain of USP11 to further investigate the pattern of DMB binding to and inhibiting USP11. DMB favorably interacts with several residues including Met776, Leu777, Glu779 and Arg886 (Figure 6A). Next, we performed site-directed mutagenesis at Met776, Leu777, Glu779 and Arg886 residue with alanine (Figure 6B) and transfected wildtype Flag-USP11 and Flag-USP11 with mutations (M776A, L777A, E779A and R886A) to the 293T cells. Streptavidin magnetic beads coupled with Biotin-DMB were incubated with 293T cell lysates. Western blot was applied to analyze the precipitates with the anti-Flag antibody. The result showed that M776A mutation decreased DMB binding to USP11 (Figure 6C). Furthermore, DMB disrupted the deubiquitination of wildtype USP11 but not USP11 with M776A mutation on GREM1 (Figure 6D). Together, these results illustrated that Met776 was vital for DMB binding to USP11 and the disruption of USP11 deubiquitinating GREM1 by DMB.

### 2.7. DMB Exhibits Preferable Antifibrotic Efficacy without Obvious Toxicity

**Figure 7:**
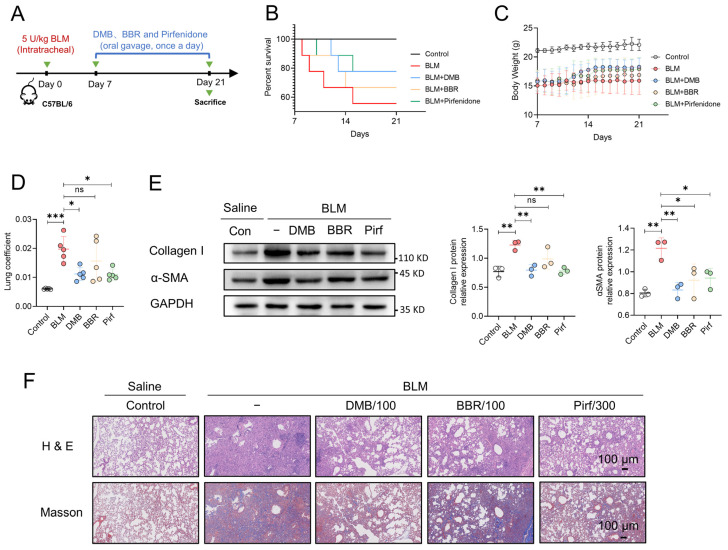
The comparison of anti-fibrotic efficacy between DMB, BBR and pirfenidone. (**A**) The schematic diagram of BLM-induced pulmonary fibrosis and treatment regimen with DMB, BBR and pirfenidone. (**B**) Survival percentage. (**C**) Body weight. (**D**) Lung coefficient of mice (n = 5) in the indicated groups. (**E**) Collagen I and α-SMA expression in the lung sections by Western blot. (**F**) Representative images of HE and Masson staining of lung tissues in different groups. Scale bar, 100 μm. Data are presented as the mean ± SD. * *p* < 0.05, ** *p* < 0.01 and *** *p* < 0.001, ns, not significant.

**Figure 8:**
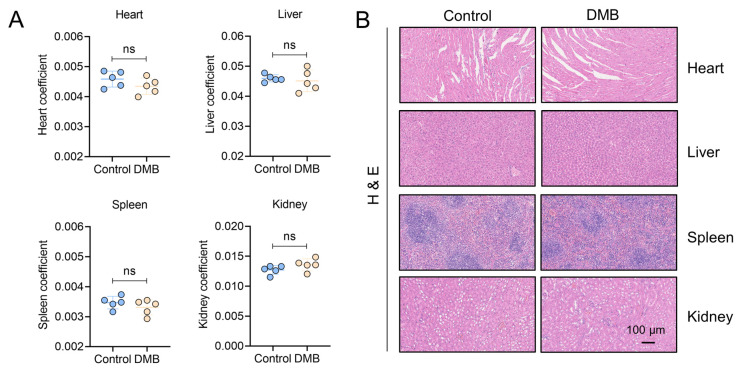
Toxicity evaluation of DMB. (**A**) The coefficients of heart, liver, spleen and kidney of mice administered 200 mg/kg DMB for 14 days (n = 5). (**B**) Heart, liver, spleen and kidney of mice administered 200 mg/kg DMB for 14 days were stained by HE. Scale bar, 100 μm. Data are presented as the mean ± SD. ns, not significant.

To evaluate the antifibrotic efficacy of DMB, berberine (BBR) and pirfenidone, BLM-induced pulmonary fibrosis was established and 100 mg/kg DMB, 100 mg/kg BBR and 300 mg/kg pirfenidone were administrated intragastrically for 14 days (Figure 7A). DMB and pirfenidone had a better effect on improving the survival situation and decreasing the lung coefficient of mice than BBR (Figure 7B–D). Western blot analysis indicated that DMB and pirfenidone decreased α-SMA and collagen I more efficiently than BBR (Figure 7E), consistently with the results of a histopathological examination, which indicated that treatment with DMB and pirfenidone improved lung morphology and reduced collagen deposition more significantly (Figure 7F). To investigate the toxicity of DMB, normal mice were administrated 200 mg/kg DMB intragastrically for 14 days. The coefficients of heart, liver, spleen and kidney were not significantly changed and there was no obvious morphology change or damage in these tissues after DMB administration (Figure 8A,B). Overall, DMB had a better effect than BBR at the same dose and equivalent effect with pirfenidone at a lower dose, suggesting that DMB exhibits preferable antifibrotic efficacy without obvious toxicity.

## 3. Discussion

Currently, the intricate pathogenesis of pulmonary fibrosis poses a challenge in fully understanding this lethal disease, while the limited availability of only two approved medications with side effects and high costs adds urgency to the search for safer and more cost-effective therapeutic alternatives. Demethyleneberberine (DMB), one of the main metabolites of berberine in vivo [44], has been demonstrated to have various anti-inflammatory, antioxidant, antifibrosis and anti-cancer bioactivities [34,36,37,38,39,45,46]. These multifarious bioactivities of DMB provide the possibility for treatment of pulmonary fibrosis. This study aimed to investigate the therapeutic potential of DMB in alleviating pulmonary fibrosis and explore the underlying mechanisms. Our work showed that DMB improved the survival state of mice, reduced pulmonary collagen deposition and improved lung tissue morphology.

During the process of EMT, epithelial cells lose their cell polarity, their connection to the basement membrane and other epithelial phenotypes, change their morphology and obtain some interstitial cell characteristics [47]. Normal wound healing of lungs undergoes the formation of temporary stroma, myofibroblast migration and wound contraction, leading to repair of barrier integrity and subsequent epithelial regeneration, remodeling and clearance of debris and the extracellular matrix [48]. However, in IPF, repeated epithelial cell damage elicits and induces abnormal repair responses, in which hyperplastic changes occur in alveolar epithelial cells and profibrotic cytokines are released, especially TGF-β, inducing an epithelial–mesenchymal transition in alveolar epithelial cells, followed by excessive accumulation of the extracellular matrix [1,49]. In addition, the transition of fibroblasts to myofibroblasts (FMT) represents an important source of myofibroblasts and its blocking contributes to pulmonary fibrosis inhibition [42,50]. Our results showed that DMB inhibited EMT and FMT in vitro. Both in vivo and in vitro studies demonstrated that DMB exhibited antifibrotic therapeutic effects, offering a new therapeutic strategy for clinical IPF management.

GREM1 is one of the major BMP antagonists [51] and aberrant expression of GREM1 contributed to pulmonary fibrosis [14,15,16,17,18]. RNA-seq was applied to analyze gene expression profiling in the lung tissues of mice in order to explore the molecular mechanism of DMB alleviating pulmonary fibrosis. Meanwhile, differential gene expression between healthy individuals and IPF patients was also analyzed. Our results indicated that GREM1 was significantly upregulated in IPF patients and mice with pulmonary fibrosis and downregulated after DMB treatment. Although GREM1 protein expression level was significantly decreased after DMB treatment, the mRNA expression level was only significantly downregulated in TGF-β1 induced A549 cells at 48 h without change at 12 h or 24 h after DMB treatment. These results indicated that DMB may also participate in other pathways to downregulate GREM1 protein expression. To investigate the mechanism by which DMB promoted GREM1 degradation, we focused on the primary pathway for protein degradation in eukaryotes, the ubiquitin-proteasome system (UPS) [21,22]. The results showed that DMB decreased GREM1 by promoting GREM1 ubiquitination. However, no studies have yet elucidated the regulatory mechanisms governing GREM1 ubiquitination in biological processes. Ubiquitination is mediated by ubiquitin-activating enzyme (E1), ubiquitin-conjugating enzyme (E2), and ubiquitin ligase (E3) for protein degradation, whereas deubiquitinating enzymes (DUBs) are able to remove ubiquitin chains from polyubiquitinated substrates for protein stabilization [52,53]. USP11 belongs to the USP family and is involved in various biological processes. It has been reported that USP11 is implicated in tumorigenesis [54,55,56], but nowadays increasing evidence indicates that USP11 is also associated with fibrosis progression by deubiquitinating EGFR or transforming growth factor β receptor II [28,29,57,58]. Our results demonstrated that USP11 colocalized with GREM1 and stabilized GREM1 by deubiquitination. Therefore, it is highly possible that DMB promotes GREM1 ubiquitination and degradation by inhibiting USP11 function. We applied the biotin–avidin system and demonstrated that DMB directly bound to USP11 and inhibited USP11 deubiquitination, resulting in GREM1 degradation. Further research revealed that Met776 of USP11 was critical for DMB binding to USP11. 

Considering that DMB is one of the main metabolites of berberine (BBR) in vivo [44] and that pirfenidone is one of only two medications approved by the FDA for IPF therapy [10], it is necessary to compare the antifibrotic activity of DMB, BBR and pirfenidone. Our results demonstrated that DMB had a better effect than BBR at the same dose and equivalent effect with pirfenidone at a lower dose, which meant much less financial burden and toxicity for IPF patients. 

## 4. Materials and Methods

### 4.1. Animals

Male C57BL/6 mice (18–20 g) were purchased from Skbex Biotechnology (Henan, China). The study protocol was approved by the Institutional Animal Care and Use Committee of China Pharmaceutical University (Animal Ethics Approval No. 2020-12-003). Mice in the control group or DMB group (mice were administered 200 mg/kg DMB daily for two weeks by oral gavage without bleomycin exposure) were administrated 50 μL sterile saline by intratracheal instillation. Mice in other indicated groups were intratracheally injected with a single dose of 5 U/kg bleomycin and were administered DMB (50, 100 and 200 mg/kg), BBR (100 mg/kg) or pirfenidone (300 mg/kg) daily for two weeks by oral gavage [35] one week after bleomycin instillation. Following the completion of drug administration, mouse tissues were harvested, weighed, and the coefficients were calculated as the ratio of tissue weight to body weight.

### 4.2. Cell Culture

MRC-5 cells were cultured in Minimum Essential Medium, A549 cells in RPMI 1640 Medium and 293T cells in Dulbecco’s Modified Eagle Medium. Ten percent FBS was added into above medium and cells were cultured at 37 °C with 5% CO2.

### 4.3. Western Blot

Cells or lung tissues were lysed to obtain total protein, the concentration of which was detected with BCA (E112-02, Vazyme, Nanjing, China). SDS-PAGE was used to separate the total protein before it was transferred to PVDF membranes. After that, the membranes were incubated with BSA and subjected to primary antibody immunoblotting. Supplementary Table S1 contains details regarding the antibodies. 

### 4.4. Immunofluorescence

A549 or MRC-5 cells cultured in 24-well cell culture plates were fixed with paraformaldehyde (4%) for 10 min. 0.5% Triton X-100 was added into PBS, which was used to incubate with cells. Cells were then blocked with PBS containing 5% BSA and 0.05% Triton X-100 and subsequently incubated with the indicated antibodies overnight at 4 °C. Coralite488 IgG were used to target above antibodies. DAPI was used to stain the nucleus.

For colocalization of GREM1 with USP11, cells were incubated simultaneously with anti-GREM1 and anti-USP11 antibodies. Alexa Fluor 488 anti-rabbit IgG and Cy3 anti-mouse IgG were applied to target primary antibodies. Supplementary Table S1 contains details regarding the antibodies.

### 4.5. Reactive Oxygen Species (ROS) Detection

To assess the ability of antioxidation of DMB, ROS was detected in TGFβ1-induced A549 cells. 

For fluorescence imaging, A549 cells were treated with TGFβ1 and DMB. After 24 h, cells were fixed with paraformaldehyde (4%) and DCFH-DA (10 μM) was then added for 30 min at 37 °C. The nucleus was stained with DAPI for 20 min. Fluorescence microscopy was used to visualize cellular ROS. 

For flow cytometry analysis, A549 cells were treated with TGFβ1 and DMB. After 24 h, cells were collected into tubes and incubated with DCFH-DA. Cellular ROS was analyzed by flow cytometry.

### 4.6. Hydroxyproline, GSH and MDA Assay

The content of GSH, hydroxyproline and MDA from lung homogenate were tested using hydroxyproline, GSH and MDA kits (Jiancheng Bioengineering, Nanjing, China).

### 4.7. ELISA

TGF-β in the lung homogenate was tested with mouse TGF-β ELISA kit (Mlbio, Shanghai, China).

### 4.8. Cell Migration

For cell migration assay, RPMI 1640 with 10% FBS was added into lower chambers. Cell suspension in RPMI 1640 without FBS was added into upper chambers with 8 μm pore size. Cells were incubated with TGF-β1 with or without DMB for 24 h. Next, chambers were washed with PBS and paraformaldehyde (4%) was used to fix cells in chambers. After removing paraformaldehyde, chambers were washed and stained with crystal violet, which was observed and captured under microscopy.

### 4.9. Immunoprecipitation

Cell Lysis Buffers (P0013, Beyotime Biotechnology, Shanghai, China) were used to extract the total protein. BCA Kit was used to detect protein concentration. The indicated antibody or control IgG were incubated with 20 μL magnetic beads (P2108, Beyotime Biotechnology, Shanghai, China) for 60 min at room temperature. The magnetic beads were washed and incubated with the cell lysates overnight at 4 °C. The beads were collected and the precipitates were obtained. Western blot was applied to analyze the precipitates.

### 4.10. In Vivo Ubiquitination Assay

The cells (293T or A549) were transfected with indicated plasmid constructs. Before harvesting, MG-132 (10 μM) was added and incubated with cells for 6 h. Next, the cells’ lysates were used for immunoprecipitation to isolate ubiquitinated GREM1. Western blot was applied to analyze the ubiquitination with anti-HA or anti-ubiquitin antibody.

### 4.11. RNA Sequencing and Bioinformatic Analysis

Lung tissues from control, BLM and BLM+DMB groups (n = 3) were used to extract total RNA. RNA-seq was performed by the Illumina NovaSeq 6000 (Illumina, CA, USA) after RNA integrity detection and sequencing libraries construction. After data quality control, reads mapping to the reference genome (GRCm38/mm10), counting the reads numbers mapped to each gene and FPKM calculation, differential gene expression was analyzed with the DESeq2 R package. Significantly differential gene expression was defined with the threshold of padj < 0.05 and |log_2_(foldchange)| > 1.

GSE110147 (Gene Expression Omnibus, GEO) contains the expression profiling of 22 IPF patients and 11 non-IPF controls. The differential gene expression between IPF patients and non-IPF controls were analyzed by R. Briefly, log2 transformation and Quantile normalization were applied to raw data. After that, differential gene expression was analyzed with the limma R package. Significantly differential gene expression was defined with the threshold of padj < 0.05 and |log_2_(foldchange)| > 1.

### 4.12. CRISPR/Cas9 Plasmid Construction

Small guide RNA sequences were designed for *USP11*-KO:

SgRNA 1#-F: CACCGAGATAGAAAACGGCGAGAGT
SgRNA 1#-R: AAACACTCTCGCCGTTTTCTATCTC
SgRNA 2#-F: CACCGTGGGCGAGAACGTCCACTG
SgRNA 2#-R: AAACCAGTGGACGTTCTCGCCCAC

Complementary oligonucleotides were annealed for 30 min at 37 °C, 5 min at 95 °C and subsequently cooled down for 15 min to 25 °C. After that, annealed primers were cloned into LentiCRISPRv2 digested by BsmBI restriction enzyme (R0739S, NEB) through ligation with T4 DNA ligase (2011A, Takara, Tokyo, Japan).

### 4.13. Synthesis of Biotin-Conjugated DMB (Biotin-DMB)

DMB (85.6 mg) and K_2_CO_3_ (109.4 mg) in anhydrous CH3CN were stirred and heated to 85 °C. After that, the linker (80 mg, CAS No.: 206265-94-3) was added and continuously stirred at 85 °C for 4 days. The reaction mixture was subsequently evaporated. The solid mixture was then washed and dried to obtain the intermediate. The intermediate (60 mg) was mixed with EDCI (17.55 mg), HOBT (12.37 mg), DIPEA (37.2 μL) and D-biotin (37.3 mg) in DMF (15 mL) at room temperature with continuous stirring. A total of 500 mL ddH_2_O was added into the reaction solution, followed by extraction with ethyl acetate and evaporation. Black solid mixture dissolved out and CH_2_Cl_2_ (1 mL) was added to dissolve it, which was subsequently separated with silica gel plate (developing solvent, CH_2_Cl_2_:Methanol:TEA = 20:1:0.05). Biotin-DMB was obtained as an orange solid compound. The structure of Biotin-DMB was illustrated by 1H-NMR and MS. The purification of Biotin-DMB was detected by High Performance Liquid Chromatography.

^1^H NMR (600 MHz, DMSO) δ 7.47, 7.41, 7.40, 7.37, 7.27, 7.26, 7.02, 6.89, 6.76, 6.72, 4.88, 4.87, 4.65, 4.64, 4.63, 4.19, 4.05, 4.04, 4.03, 4.02, 4.00, 3.99, 3.91, 3.90, 3.89, 3.88, 3.83, 3.80, 3.79, 3.78, 3.76, 3.65, 3.63, 3.62, 3.60, 3.52, 3.51, 3.07, 3.05, 2.30, 2.29, 2.27, 2.23, 2.22, 2.20, 2.18, 2.17, 2.03, 2.01, 1.99, 1.91, 1.66, 1.51, 1.50, 1.48, 1.39, 1.37, 1.36, 1.35, 1.34, 1.34, 1.30, 1.26, 1.25, 1.19, 1.18, 1.17, 1.15.

### 4.14. Biotin-DMB Binding Assay

A total of 20 μL streptavidin magnetic beads (P2151, Beyotime Biotechnology, Shanghai, China) were incubated with Biotin-DMB (200 μM) or Biotin (200 μM) for 2 h at room temperature. The streptavidin magnetic beads coupled with Biotin-DMB or Biotin were then incubated with cell lysates overnight at 4 °C. The beads were collected and the precipitates were obtained. Western blot was applied to analyze the precipitates.

### 4.15. Site-Directed Mutagenesis

Full-length USP11 was amplified from human cDNA by PCR and sub-cloned to pcDNA3.1 plasmid after digestion with HindIII (1615, Takara) and EcoRI restriction enzyme (1611, Takara) and ligation with T4 DNA ligase (2011A, Takara). Site-directed mutagenesis at residue Met776, Leu777, Glu779 and Arg886 was conducted, respectively, by PCR with the following primers.

Met776 F: CTGTGGGCACTGCCGGAGATTCTCATCATCCACCTGAAACGC
Met776 R: CGGCAGTGCCCACAGGTCCAGCTTCTTGGTTGCCAGCTGGTG
Leu777 F: TGGATGGCACCGGAGATTCTCATCATCCACCTGAAACGCTTT
Leu777 R: CTCCGGTGCCATCCACAGGTCCAGCTTCTTGGTTGCCAGCTG
Glu779 F: CTGCCGGCAATTCTCATCATCCACCTGAAACGCTTTTCCTAC
Glu779 R: GAGAATTGCCGGCAGCATCCACAGGTCCAGCTTCTTGGTTGC
Arg886 F: TACCAAGCACAGGACGTGGCGCGACGCCTGCTGTCCCCGGCC
Arg886 R: GTCCTGTGCTTGGTAGAAGAGGACATAGGCTGCCTTGGACTC

### 4.16. Molecular Docking

The protein structure of USP11 was downloaded from AlphaFold Protein structure Database (UniProt ID: G5E9A6). The catalytic domain of USP11 was used to perform docking with DMB by Molecular Operating Environment (MOE 2020.09, Chemical Computing Group Inc., Montreal, QC, Canada).

### 4.17. Quantitative Real-Time PCR

Total RNA was extracted and used to generate cDNA under All-In-One RT MasterMix kit protocol (G490, Abm, Vancouver, Canada). SYBR green qPCR MasterMix (B21202, Bimake, San Diego, CA, USA) was used for qRT-PCR. GAPDH was applied as a reference gene. Expression data of the target genes were calculated with the 2^−ΔΔCt^ method. The primer sequences are as follows:

*GREM1* F: CGGAGCGCAAATACCTGAAG
*GREM1* R: GGTTGATGATGGTGCGACTGT
*GAPDH* F: ACAACTTTGGTATCGTGGAAGG
*GAPDH* R: GCCATCACGCCACAGTTTC

### 4.18. Statistical Analysis

Two-tailed Student’s *t* test for two groups or One-way ANOVA for three or more groups were applied to analyze the statistical significance. *p* < 0.05 was considered statistically significant. All data were presented as mean ± SD in this study.

## 5. Conclusions

In conclusion, we reported that DMB alleviated pulmonary fibrosis by promoting GREM1 degradation through Ub/proteasome pathway. USP11 was revealed to colocalize with GREM1 and stabilize GREM1 by deubiquitination. Furthermore, it was demonstrated that DMB selectively bound to the Met776 residue of USP11, leading to the inhibition of USP11 and disruption of USP11-deubiquitinating GREM1. In addition, DMB showed an equivalent therapeutic effect at a lower dose without obvious side effects compared with pirfenidone, exhibiting preferable antifibrotic efficacy and high safety for treatment of pulmonary fibrosis.

## Data Availability

The RNA-seq data are available in the Gene Expression Omnibus (GEO) database (GSE226924, https://www.ncbi.nlm.nih.gov/geo/query/acc.cgi?acc=GSE226924 (accessed on 18 February 2024)). The other data supporting the findings in this study are available within the article and the Supporting Information files.

## Supplementary Materials

The following supporting information can be downloaded at: https://www.mdpi.com/article/10.3390/ph17030279/s1. Table S1: Antibody information. Figure S1: Differential expression genes analysis of homo sapiens and mice. Figure S2: Structure verification and purification analysis of Biotin-DMB. Figure S3: Protein structure of USP11 from AlphaFold Protein structure Database (UniProt ID: G5E9A6).

## Abbreviations

BCA: Bicinchoninic Acid Assay; BLM, Bleomycin; BSA, bovine serum albumin; DMB, Demethyleneberberine; ECM, extracellular matrix; ELISA, enzyme-linked immunosorbent assay; EMT, epithelial–mesenchymal transition; FMT, fibroblast–myofibroblast transition; GAPDH, Glyceraldehyde-3-phosphate dehydrogenase; GREM1, Gremlin-1; GSH, glutathione reduced; H&E, hematoxylin-eosin staining; IPF, Idiopathic pulmonary fibrosis; Masson, Masson’s trichrome staining; MDA, Malondialdehyde; MS, mass spectrum; PBS, phosphate buffer saline; qRT-PCR, Quantitative Real-Time PCR; ROS, reactive oxygen species; TGFβ1, transforming growth factor β1; Ub, ubiquitin; USP11, ubiquitin-specific protease 11.
