# Supplementary material for: Demethyleneberberine Alleviates Pulmonary Fibrosis through Disruption of USP11 Deubiquitinating GREM1
Source: Pharmaceuticals (Basel). 2024 Feb 22;17(3):279. doi: 10.3390/ph17030279 (PMC10974697; doi:10.3390/ph17030279)
Supplement: Supplementary file 1 [file pharmaceuticals-17-00279-s001.zip › pharmaceuticals-2840664-supplementary.pdf]

Supplementary Material

Demethyleneberberine Alleviates Pulmonary Fibrosis through Disruption of USP11 Deubiquitinating GREM1

Chuang Ge, Mengsheng Huang, Yanhong Han, Chang Shou, Dongyin Li and Yubin Zhang *

Contents in Supplementary Material

Table S1, Figure S1-S3 and Figure legends

Table S1. Antibody information

Antibodies	Sources	Cat#	WB	IF	IHC
GAPDH	Proteintech	60004-1-Ig	1:10000		
Collagen I	Proteintech	67288-1-Ig	1:20000	1:200	1:5000
α-SMA	Proteintech	14395-1-AP	1:5000	1:200	1:3000
Vimentin	Santa Cruz	sc-6260	1:1000		
Fibronectin	Proteintech	66042-1-Ig	1:10000	1:200	
GREM1	Santa Cruz	sc-515877	1:1000		
USP11	Proteintech	22340-1-AP	1:4000		
Ubiquitin	Proteintech	10201-2-AP	1:1000		
Myc	Proteintech	16286-1-AP	1:4000		
Flag	Sigma	F1804	1:1000		
HA	Proteintech	66006-2-Ig	1:20000		
c-IAP2	Wanleibio	WL01254	1:500		
ALK5	Wanleibio	WL03150	1:500		
IκBα	Wanleibio	WL01936	1:500		
Coralite488 anti-rabbit IgG	Proteintech	SA00013-2		1:500	
Coralite488 anti-mouse IgG	Proteintech	SA00013-1		1:500	
Cy3 anti-mouse IgG	Invitrogen	A10521		1:200	
Alexa Fluor 488 anti-rabbit IgG	Jackson ImmunoResearch	111-545-144		1:200	

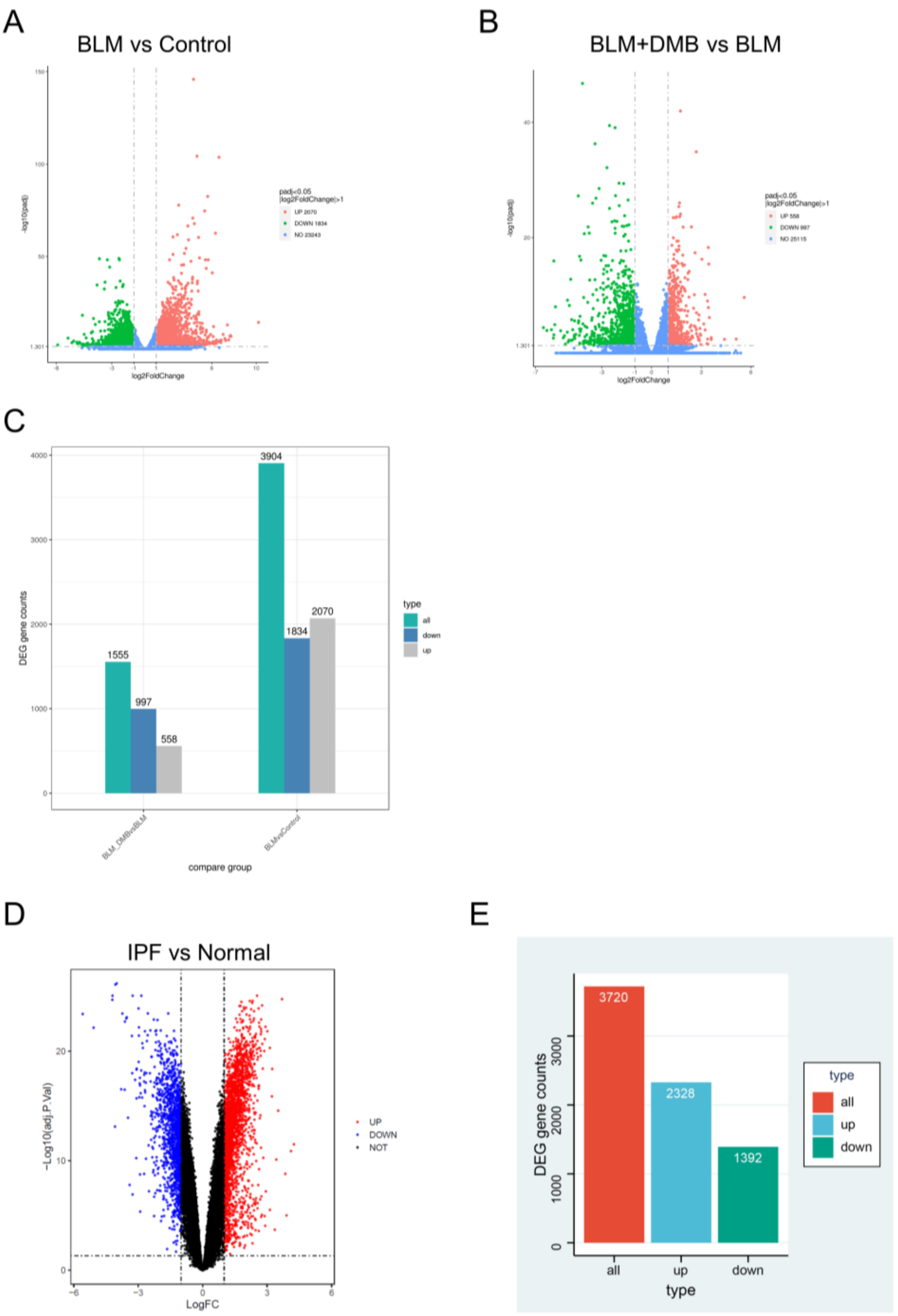

Fig. S1. Differential expression genes analysis of homo sapiens and mice. (**A-C**) Differential expression genes analysis of mice, volcano plot of BLM vs Control (**A**) and BLM + DMB vs BLM (**B**), numbers of differential expression genes (**C**). (**D, E**) Differential expression genes analysis of homo sapiens, volcano plot of IPF vs Normal (**D**), numbers of differential expression genes (**E**).

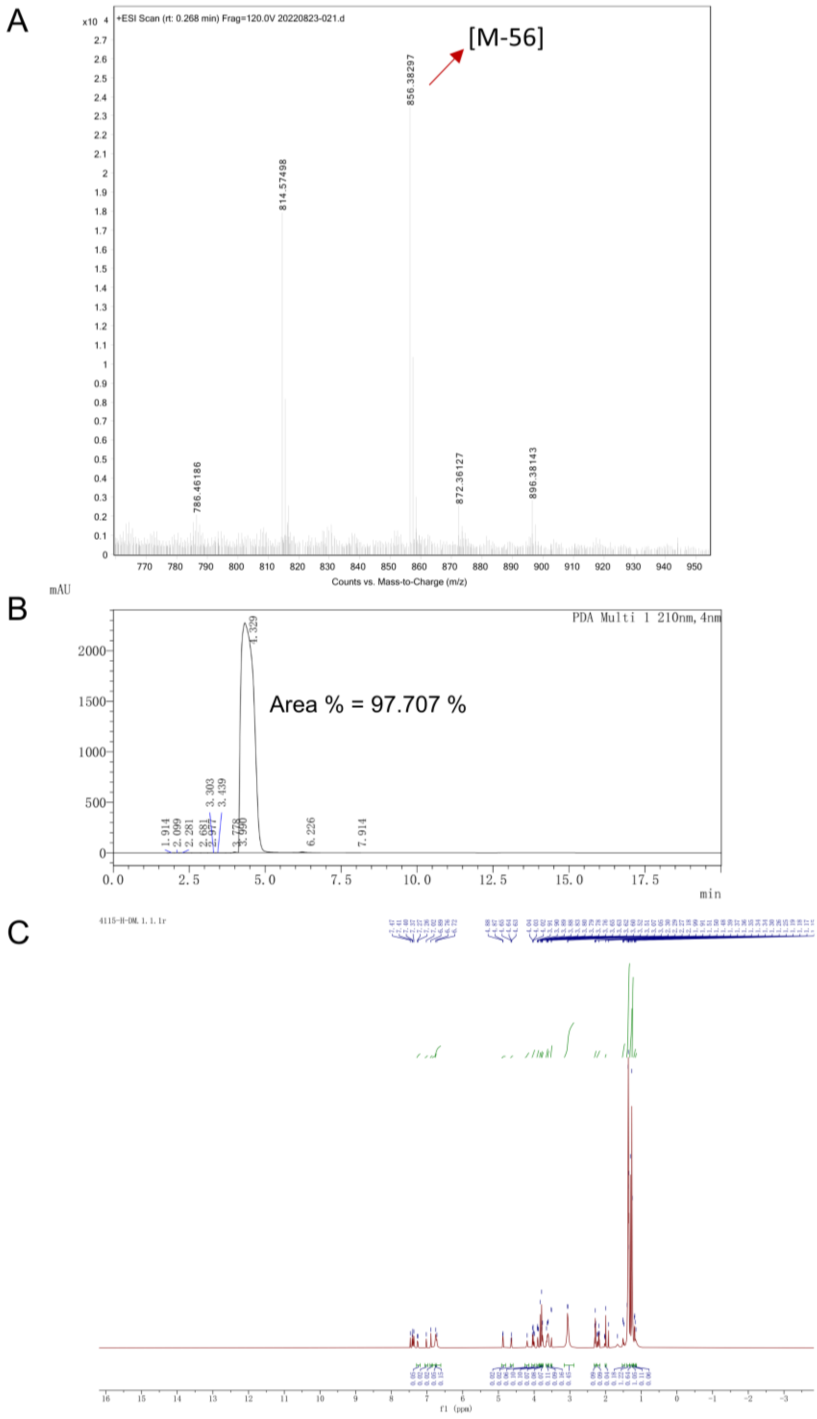

Fig. S2. Structure verification and purification analysis of Biotin-DMB. (**A**) Mass spectrum of Biotin-DMB. (**B**) Purification analysis of Biotin-DMB. (**C**) H-NMR of Biotin-DMB.

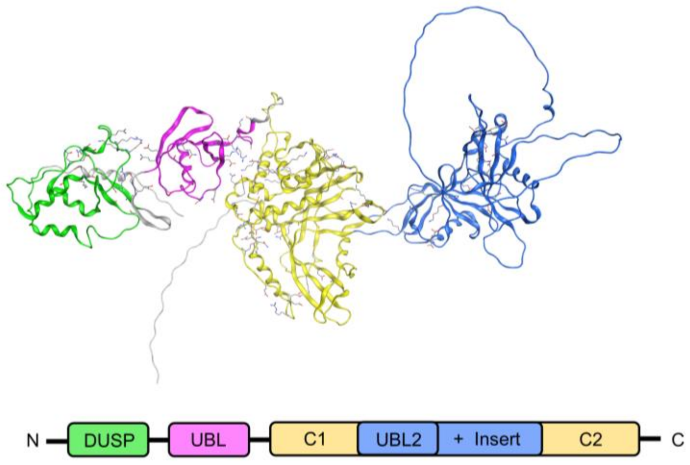

Fig. S3. Protein structure of USP11 from AlphaFold Protein structure Database (UniProt ID: G5E9A6).
